# The effectiveness and safety of pharmaceuticals to manage excess weight post-bariatric surgery: a systematic literature review

**DOI:** 10.1080/21556660.2019.1678478

**Published:** 2019-10-17

**Authors:** Sean Wharton, Elham Kamran, Mahnoor Muqeem, Amina Khan, Rebecca A. G. Christensen

**Affiliations:** a The Wharton Medical Clinic, Toronto, ON, Canada;; b Kiniesiology and Health Science, York University, Toronto, ON, Canada

**Keywords:** Bariatric surgery, weight loss, pharmaceutical, weight regain

## Abstract

**Objective:** To systematically review the literature on weight management pharmaceutical use in patients who have had bariatric surgery.

**Methods:** Google Scholar, Pubmed, Cochrane, Embase, Web of Science, and Clinical Trials were searched from inception to December 31st, 2018 inclusive.

**Results:** Thirteen studies met inclusion and reported decreases in weight with the use of weight management medications in post-bariatric surgical patients. Five studies examined weight loss outcomes by the type of bariatric surgery procedure, and four of these studies observed less weight loss in patients who had undergone gastric sleeve compared to those who had roux-en-y bypass (*n* = 3 papers) and adjustable gastric banding (*n* = 1 paper) with medication use. Four studies compared the effectiveness of medications for weight management and observed slightly greater weight loss with the use of topiramate and phentermine as a monotherapy compared to other weight loss medications. Using a sub-sample of participants, authors observed less weight loss on metformin but not phentermine or topiramate for younger adults. Another post-hoc analysis in the same sample observed greater weight loss for older adults with liraglutide 1.8 mg. Side effects were reported in seven studies and were overall consistent with those previously reported in non-surgical populations.

**Conclusion:** Results of this systematic review suggest pharmacotherapy may be an effective tool as an adjunct to diet and physical activity to support weight loss in post-bariatric surgery patients. However, due to most studies lacking a control or placebo group, more rigorous research is required to determine the efficacy of this intervention.

## Introduction

Bariatric surgery is the gold standard obesity treatment. Evidence shows that bariatric surgery is associated with greater weight loss in comparison to nonsurgical treatments^1^. However, 10–20% of the post bariatric surgery patients regain a significant amount of their lost weight^1^. There are limited treatment options for individuals with excessive weight regain or insufficient weight loss, and primarily consist of lifestyle interventions or additional surgeries^2^^,^^3^. Due to the exclusion of post-bariatric surgery patients from weight loss pharmaceutical phase trials, currently weight loss medications are not approved for use in this population. However, there is a growing body of literature examining the off-label prescription of weight loss medications by physicians. Therefore, it appears that despite the lack of regulatory approval, physicians may be prescribing weight loss pharmaceuticals to post-bariatric surgery patients. This suggests there is a need to synthesize the available evidence to support clinicians and patients who have had bariatric surgery in making informed decisions regarding the use of weight loss medications. The objective of this study is to systematically evaluate the available literature on the use of weight management pharmaceuticals in post-bariatric surgery patients with excessive weight regain or insufficient weight loss.

## Methods

This reporting of this systematic review was done in accordance with the Preferred Reporting Items for Systematic Review and Meta-Analysis (PRISMA) statement. All criteria were determined a-priori.

Google Scholar, Pubmed, Cochrane, Embase, Web of Science, and Clinical Trials were searched from their inception to December 31st, 2018 for articles that met inclusion. The search was conducted to identify articles which examined patients who had initiated pharmaceuticals to support weight loss after they had undergone bariatric surgery and search terms were altered as necessary for each database.

For Google Scholar the following search terms were used: orlistat OR liraglutide OR sibutramine OR phentermine AND topiramate OR naltrexone AND bupropion OR lorcaserin OR weight loss medication AND [(post- OR after AND bariatric OR metabolic OR Roux-En-Y OR Sleeve OR Band OR Biliopancreatic) AND surgery]. The search was restricted to exclude patents and citations, and to include all articles published to December 31, 2018 inclusive.

Two authors (AK and MM) compiled the list of returned articles into excel. Titles and abstracts were screened, and full papers were then evaluated for eligibility. If there was a disagreement on whether an article should be included, a third author (RAGC) made the final determination.

Articles met inclusion for this review if they satisfied all of the following criteria: (1) conducted in humans; (2) conducted in adults; (3) population had undergone weight loss surgery; (4) subjects were prescribed a pharmaceutical for weight loss post-bariatric surgery; (5) estimate of change in weight, or data to calculate change in weight; (6) peer-reviewed; (7) reported in English. Articles were excluded if they examined nonrestrictive/cosmetic types of weight loss surgery (e.g. liposuction). No other restrictions were applied to the search.

Population characteristics, study type, type of the bariatric surgery, medication type, change in weight, side effects, and duration on medication were extracted independently by two authors (AK and MM) from each article into excel and evaluated for discrepancies by a third author (RAGC).

Variables were extracted as is where possible. However, one study provided patient level data but no mean change in weight. This was the study by Zilberstein et al.^4^. In this case, the mean postoperative weight of all 16 patients was calculated (104.1 kg) and this value was subtracted from the mean post-topiramate weight (97.0 kg).

The quality of included articles were assessed by two authors (RAGC and EK) using a quality assessment tool for quantitative studies^5^^,^^6^. If there was a discrepancy in the scoring, the authors rereviewed the criteria for where there was a discrepancy (e.g. blinding, confounders), and then independently rerated that section. In the case there was still a discrepancy, the scoring for that section was determined by consensus among the two authors.

## Results

**Figure 1.**
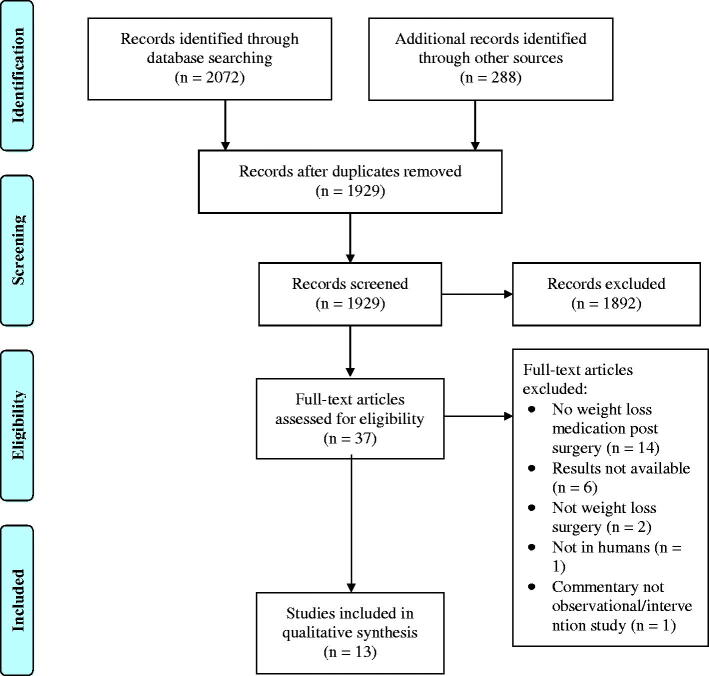
PRISMA Flow-chart.

Of the 1929 articles screened, 37 fit the criteria for assessment and were assessed in full. Articles were excluded if the study did not examine the use of weight loss medication post-bariatric surgery (*n* = 14), did not have results available either due to early phase of study or just not reported (*n* = 6), used animal subjects (*n* = 1), reported nonrestrictive (e.g. sclerotherapy) or cosmetic procedures (e.g. liposuction) for weight loss (*n* = 2), or were not available in full-text (*n* = 1) (Figure 1). Thirteen articles satisfied all inclusion criteria, two of which were post-hoc analysis^7^^,^^8^ which examined a subset of patients included in the original study by Stanford et al (2017)^9^. Characteristics of studies that met inclusion can be found in Table 1. This appears to correspond to 931 unique patients examined, with individual study sample sizes ranging from 3^18^ to 319^9^ participants. Patients ranged in age from 20 to 73 years.

**Table 1.** Characteristics of studies which met inclusion.

Author	Population Characteristics	Study Type	Type of Bariatric Surgery	Duration	Weight Management Medication	Treatment initiation in relation to the surgery; Indication for initiating the weight loss medication	Weight Effect
Gorgojo-Martinez (2016)^15^	164 M/FBMI: Bariatric surgery patients 40.3 ± 9.2 kg/m^2,^; Non-bariatric surgery patients39.7 ± 7.6 kg/m^2^Mean age 53.0 ± 9.0 years	Retrospective cohort	RNY (53.3%), BPD (20%), Vertical Gastric Banding (13.3%), SG (6.7%) and AGB (6.7%)	104 weeks	Liraglutide 1.8mg	NDFailure to lose weight in the previous year despite a structured lifestyle program. A weight gain of >5% of the minimum weight reached post bariatric surgery was considered relevant	There were similar significant decreases in weight for the subjects who had bariatric surgery (3.4kg, *p* =.011) and those who did not (2.3kg, *p* =.011).
Guerdjikova (2005)^18^	3 F44–55 years35.3 kg/m^2^	Case series	RNY	4 to 17 months (mean: 30 months)	Topiramate	14-30 months post-surgeryReturn of binge eating and weight regain (5-20 kg).	All patients lost weight (range: 2 to 31.7kg, mean: 16.1 kg, *P-value* NR).
Nor Hanipah (2018)^13^	209 M/FBMI: NRMean age: 50.9 ± 10.5 years	Retrospective cohort	RNY (60.3%), SG (24.9%), ABG (10.0%), Gastric plication (1.9%), and revisional surgery (2.9%)	3 months to 12 months	Phentermine, phentermine/topiramate, lorcaserin, naltrexone/bupropion	Median time of 38 months (interquartile range, 24-63)Weight regain (>5% nadir) or poor weight loss (<50% excess weight loss) after bariatric surgery.	Patients lost 3.2% of their body weight by 3 months and 2.4% by 12 months (*P-value* NR). Patients with AGB (4.6%) and RNY (2.8%) lost significantly more weight than those with SG (0.3%, *p≤*.02).
Jester (1996)^11^	34 M/FBMI: 34.0 kg/m^2^Age range: 27-65 years	Retrospective cohort	RNY (35.3%), and BPD (64.7%)	12 weeks	Phentermine and fenfluramine as a monotherapy or in combination	≥18 months post-operative ≥9 kg above ideal body weight	All patients lost weight (4.5 to 22.7 kg, *P-value* NR). RNY lost on average 11.4 kg and BDP 11.8 kg (*P-value* NR).
Pajecki(2012)^12^	15 M/FBMI: 42.4 kg/m^2^Mean age: 47.2 ± 12.5 years	Retrospective cohort	AGB (26.6%), RNY (60.0%), Duodenal switch BPD (6.7%), and SG (6.7%)	8 to 28 weeks (mean: 12.5 weeks)	Liraglutide 1.8mg	The mean postoperative period was 5.6 years, Range: 2-13 years<50% of excess weight loss after >2 years of follow-up, or regained ≥15% of lowest weight post-surgery	All patients lost weight (range: 2 to 18 kg, mean: 7.5 kg, *p*<.001).
Rye (2018)^14^	20 M/FBMI: NDMean age: 49.6 ± 8.3 years	Retrospective cohort	RNY (35%), SG (35%), VBG (15%), and AGB (15%)	28 weeks	Liraglutide 3.0 mg	76.3 ± 72.9 monthsWeight regain (>10%), inadequate weight loss (<20% weight loss from surgery) and plateau	At 16 weeks median percentage loss was 7.1% (IQR 5.1-12.2%), and at 28 weeks 9.7% (IQR 7.8-13.9%) (P-value for change from baseline N.R.).
Schwartz (2016)^16^	65 M/FBMI: 39.3 kg/m^2^Mean age: 47.3 ± 0.8 years	Retrospective cohort	RNY (78%), and AGB (22%)	90 days	Phentermine or combination phentermine and topiramate	The median time from minimum weight post-surgery to initiate Ph cohort was 35.3 months and for PhT cohort was 36.3 months.Patients had to have a BMI of 27–29.9 kg/m2 with at least one comorbid condition, or a BMI >30 kg/m2 without co-morbidities.	All patients lost weight. Patients taking phentermine (6.3kg) lost more weight than those taking a combination of phentermine and topiramate (3.8kg, *p* =*.025)*. Weight loss did not differ by type of surgery. (*p* = .345).
Srivastava (2018)^10^	48 M/FBMI: 38.3 kg/m^2^Age: NR	Retrospective cohort	RNY (52.0%), SG (29.2%) and AGB (18.8%)	NR	Metfromin, phentermine, phentermine/topiramate, bupropion/naltrexone, lorcaserin, zonsiamide, topiramate, and glucose-like 1 agonists	Mean time of 6.1 ± 4.6 years (range: 1-20 years)ND	Bariatric surgery patients taking no WL meds, 1 WL med, or ≥ 2 WL meds lost 2.2 kg, 2.7 kg, and 5.7 kg, respectively (change from baseline *P*-value NR, P-value for trend *p* =.05). Non-bariatric surgery patients who took 0 WL meds, 1 WL med, or ≥2 WL med lost 4.8 kg, 6.8 kg, 9.5 kg (change from baseline *p*-value NR*, p*-value for trend <.05). No significant difference in WL for those ≥ 2 meds based on bariatric surgery status (*p* =.08).
Stanford (2017)^9^	319 M/FBMI RNY: 36.8 kg/m^2^ SG: 37.5 kg/m^2^Age range: 20-73 years	Retrospective cohort	RNY (80.9%) and SG (19.1%)	NR	Phentermine, topiramate, zonisamide, metformin, bupropion, orlistat, sibutramine, liraglutide, exenatide, pramlintide, naltrexone, lorcaserin, phentermine/topiramate, canagliflozin or bupropion/naltrexone	SG: 23.2 ± 15.3 monthsRNY: 59.3 ± 36.7monthsAfter weight regain (78.5%; *n* = 249) or at plateau (21.5%; *n* = 68). Amount ND.	Patients with RNY lost 9.0 kg and SG lost 4.5 kg. Patients with RNY were more likely to lose ≥10% and ≥ 15% of their weight than those with SG (*p* ≤ .03). Patients taking topiramate (9.2 kg) lost significantly more weight than those other weight loss medications (6.4 kg, *P-value* NR).
Stanford (2018)^7^	35 M/FBMI RNY: 34.7 ± 5.8 5kg/m^2^SG: 36.6 ± 3.6 5 kg/m^2^≥60 years	Post-hoc retrospective cohort	RNYSG	NR	Phentermine, topiramate, zonisamide, metformin, bupropion, orlistat, sibutramine, liraglutide, exenatide, pramlintide, naltrexone, lorcaserin, phentermine/topiramate, canagliflozin, and bupropion/naltrexone	RNY: 38.5 ± 28.6 months SG: 26.2 ± 13.2 monthsPatients were more likely to be prescribed medications after weight regain (71.4%; *n* = 25) occurrence than at plateau (28.6%; *n* = 10)	Patients lost an average BMI of 2.74 ± 2.6 kg/m^2^ on medications. . RNY patients lost a greater BMI than SG (3.37 ± 2.83 kg/m^2^ vs. 1.38 ± 1.49 kg/m2, *p* =.0372). liraglutide more effective than the other medications. Coefficient observed for liraglutide *β* = −16.07 (*p* =.009, CI: -25.17-3.57).
Toth (2018)^8^	37 M/FBMI: RNY: 38.9 ± 9.1 kg/m^2^, SG: 37.3 ± 5.7 kg/m^2^Range: 21-30 years	Post-hoc retrospective cohort	RNY (75.7%) and SG (25.7%)	NR	Topiramate, phentermine, and/or metformin	RNY: 62.6 ± 39.1 monthsSG: 20.1 ± 5.2 months ND	RNY had higher median percent weight loss (8.1%) than SG (3.3%; *p* = .0515). Median percent weight change on metformin was less than the rest of the cohort (-2.9% vs. 7.7%; *p* =.0241). No difference from the rest of the cohort for phentermine (*p* =.2018) or topiramate (*p* = .3187)
Zilberstein (2004)^4^	16 M/FBMI: 45.3 kg/m^2^Age range: 20-44 years	Prospective cohort	Swedish AGB	90 days	Topiramate or fluoxetine	NDDid not have acceptable weight loss (acceptable ND).	Patients lost 7.1 kg on average (*p* = .0139)^a^
Zoss (2002)^17^	38 M/FBMI: 41.0 kg/m^2^Mean age: 50 ± 2.0 for AGB + DC and 47 ± 2.0 for AGB+Orlistat+DC	Matched prospective cohort	Swedish AGB	8 months	Orlistat	18 ± 6 monthsStopped losing weight ≥3 months before study start	Patients taking orlistat lost significantly more weight (8 kg) than those not prescribed orlistat (2 kg, *p*<.02).

Abbreviations. M: Male; F: Female; AGB: Adjustable Gastric Banding; VBG: Vertical Banded Gastroplasty; RNY: Roux-En-Y Gastric Bypass; BPD: Biliopancreatic Diversion; ND: Not Reported; SG: Sleeve Gastrectomy; DC: Diet counseling; SEM: Standard error of the mean.

^a^Value is calculated from postoperative weight minus post-topiramate weight using patient-level data.

There were differences in the study design of the 13 articles included in this review. Eleven of the studies were cohort studies^4^^,^^7–16^, and the remaining two are a matched prospective cohort^17^ and case series^18^. Types of bariatric surgery procedures also varied, but the most common procedure was roux-en-y gastric bypass (RNY), in ten^7–13^^,^^15^^,^^16^^,^^18^ studies, followed by adjustable gastric banding (AGB)^4^^,^^10^^,^^12^^,^^13^^,^^15–17^ and sleeve gastrectomy (SG)^7–10^^,^^12^^,^^13^^,^^15^ both in seven studies.

The medications prescribed for weight loss also varied greatly among studies. However, the majority of the studies prescribed topiramate^4^^,^^7–10^^,^^18^, and phentermine^4^^,^^9^^,^^11^^,^^13^^,^^14^^,^^16^^,^^18^ as a mono- or combination therapy^9^^,^^10^^,^^13^^,^^16^. Medication initiation in relation to surgery ranged by study and patient, from 14 months^18^ to 13 years^12^. The indication for initiating the weight loss medication varied across the studies. Some of the indications include: (1) weight regain, which was either not defined^7^^,^^9^^,^^18^ or ranged from 5 to 15%^13–15^; (2) weight plateau^7^^,^^10^; (3) failure to lose weight after bariatric surgery, which was not well defined^18^. Study durations ranged from 2 months to 24 months. Despite all the differences listed above, all thirteen studies reported decreases in weight with the use of pharmaceuticals in post-bariatric surgery patients.

Five studies^7–9^^,^^11^^,^^13^ examined weight loss outcomes by the type of bariatric surgery procedure. Four studies reported less weight loss for patients who have undergone SG compared to AGB^13^ (*p* = .02) and RNY^7–9^^,^^13^ when taking weight loss pharmaceuticals. Two of these four studies were post-hoc analysis of an original study^9^ and showed the same consistent pattern in younger (21–30 years; *p =* .05151)^8^ and older (≥60 years; *p* = .03)^7^ adults. Conversely, studies reported similar weight loss among RNY, and BDP and AGB patients’ post-initiation of weight management medication. Specifically, one study^11^ reported non-significantly different weight loss outcomes for patients with previous RNY and BPD (11.4 kg compared to 11.8 kg at 12 weeks), and another report differences in total weight loss percent at 12 months for patients with RNY (2.8%) and AGB (4.6%), respectively, but they were also not statistically significant^13^.

Five^12^^,^^14^^,^^15^^,^^17^^,^^18^ papers focused on one medication for weight loss post bariatric surgery, whereas eight^4^^,^^7–11^^,^^13^^,^^16^ papers studied the use of multiple medications. Four^7–9^^,^^16^ of these eight studies compared weight loss outcome according to type of medication. Two^9^^,^^16^ papers identified monotherapy with topiramate or phentermine to have the greatest impact on weight loss in post-bariatric surgery patients. One study reported that topiramate had greater weight loss effects compared to phentermine, metformin, bupropion and zonisamide (20.2 ± 24.5lbs versus 13.99 ± 13.6lbs, *p*-value not reported), in patients who had previous RNY or SG bariatric surgery^9^ and were twice as likely to lose at least 10% of their post-surgical weight^9^ (*p* = .018) than patients who were prescribed one of the other aforementioned medications. In the other comparative study, phentermine had greater weight loss as monotherapy, when compared to combination therapy with topiramate, (3.8 kg (95% CI: 1.08, 6.54) compared to 6.3 kg (95% CI: 4.25, 8.44), over 90 days, respectively)^16^. Two of the studies were post-hoc analysis of this original study. One study observed a lower median percent weight change on metformin than the rest of the cohort (−2.9% vs. 7.7%; *p* = .0241) in younger adults. However, there was no difference from the rest of the cohort for phentermine (*p* = .2018) and topiramate (*p* = .3187)^8^. The other post-hoc study found liraglutide 1.8 mg was more effective than the other medications in older adults (*β* = −16.07, *p* = .009, 95% CI: −25.17, 3.57)^7^.

Side effects for weight loss medications were reported in seven^4^^,^^11^^,^^12^^,^^15^^,^^17^^,^^18^ studies. These studies reported side-effects for topiramate^4^^,^^14^^,^^18^, orlistat^17^, liraglutide^12^^,^^14^^,^^15^, fenfluramine^11^, and phentermine^11^. For orlistat use, gastrointestinal (GI) side effects such as increased gas-bloating and increased stool frequency were reported^17^. Topiramate was discontinued in two patients (12.5%), who experienced drowsiness^4^. In another study, side effects reported with topiramate use included mild fatigue, transient insomnia and tingling sensation at lower doses (25–50 mg), and transient cognitive dysfunction/word finding difficulty and evening anxiety at higher doses (150–625 mg)^18^. These side effects were experienced by all three participants (100%) in the study^18^. Memory loss was reported for several patients taking phentermine, but was reversed when treatment was completed or discontinued^11^. However, the exact number of patients who experienced this side effect was not reported. In one study, side effects of liraglutide 1.8 mg were reported by only 1 patient (6.7%) and included mild transient nausea and vomiting which did not require antiemetic drugs^15^. Another study also reported nausea as a side effect of liraglutide 1.8 mg experienced by six patients (40%)^12^. However, this was controlled with temporary medication dose reduction^12^. For patients taking liraglutide 3.0 mg, nausea as the most common side effect (37.9%, *n* = 11 of 33 patients), and one patient discontinued due to GI side effects^14^. Side effects related to fenfluramine were reported in one study^11^. One patient (2.9%) demonstrated significant memory loss, and another patient (2.9%) developed a rash^11^.

The global quality rating for each article can be found is presented in Table 2. Overall, articles^7^^,^^9–16^^,^^18^ received a moderate or strong quality rating (84.6%, *n* = 11 of 13). In fact, the majority of articles received a strong rating (69.2%, *n* = 9 of 13)^7^^,^^9^^,^^10^^,^^12–16^, and only two articles^4^^,^^17^ (15.4%) received a weak rating.

**Table 2.** Global rating of quality assessment of articles.

Author	Rating
Gorgojo-Martinez^15^	Strong
Guerdjikova^18^	Moderate
Jester^11^	Moderate
Nor Hanipah^13^	Strong
Pajecki^12^	Strong
Rye^14^	Strong
Schwartz^16^	Strong
Srivastava^10^	Strong
Stanford^9^	Strong
Stanford^7^	Strong
Toth^8^	Strong
Zilberstein^4^	Weak
Zoss^17^	Weak

## Discussion

There are limited options for the management of insufficient weight loss or excessive weight regain post bariatric surgery. Hence, there is an eminent need for additional postoperative treatment options for excess weight in this population. Eleven original, and two post-hoc studies of relatively good quality have examined postoperative pharmaceutical use for weight loss and observed decreases in weight. Key findings from the review suggest that pharmacotherapy as an adjunct to diet and physical activity may be helpful to support weight loss in post-bariatric surgery patients. While there was evidence of weight loss differences based on the medication and type of bariatric surgery, patients do not appear to experience a disproportionate burden of side effects from taking these medications.

Trials for weight loss medications often exclude patients who have had bariatric surgery. As such, weight loss pharmaceuticals are not approved for use in this patient population. Similar to what is observed in non-post-surgical populations, results of this systematic literature review suggest post-bariatric surgery patients taking pharmaceuticals for weight loss can have reductions in weight^4^^,^^7–18^. While studies examined different pharmaceuticals, and surgery types, all reported weight loss.

Two studies included a comparison group; two studies^10^^,^^15^ compared patients with and without bariatric surgery, and one^10^ made comparisons among bariatric surgery patients who did and did not take weight loss pharmaceuticals. As expected, bariatric surgery patients taking weight loss medication lost more than those who were participating in a lifestyle intervention alone. In addition, there was no significant difference in weight loss based on whether the patients had bariatric surgery. Due to the dearth of research in this area, there is a need for more research with direct comparisons of post-bariatric surgery to non-post bariatric surgery patients, and to post-bariatric surgery patients prescribed a placebo to establish the efficacy of weight loss agents in post-bariatric surgery populations, which is already underway. For example, there is a randomized control trial by Miras et al (2019)^19^ published after the dates of this review. The authors compared the use of liraglutide 1.8 mg in post-bariatric surgery patients. Consistent with the observational research, those prescribed the weight management medication lost significantly more weight (4.23 (95% CI: 1.61, 6.81 kg) *p* = .0017).

Four studies^7–9^^,^^16^ have directly compared weight loss by type of medication in post-bariatric surgery patients, and observed differences in effectiveness. Stanford et al (2017)^9^ reported that taking topiramate was associated with significantly greater weight loss for post-bariatric surgery patients compared to the other medications examined (20.2 lbs versus14.0 lbs, respectively). Two post-hoc analysis of this study observed less weight loss with metformin in younger (21–30 years; *p =* .05151)^8^ adults, and greater weight loss with liraglutide 1.8 mg in older (≥60 years; *p* = .03)^7^ adults. Schwartz et al (2016)^16^ observed greater weight loss with the use of phentermine (6.35 kg) as monotherapy compared to a combination phentermine and topiramate (3.81 kg) in post-bariatric surgery patients. However, as these studies were observational, differences in treatment indication rather than medication efficacy could be the source of these differences in weight loss. Thus, there is still a need for head-to-head research to determine if certain weight management pharmaceuticals are more efficacious for post-bariatric surgery patients than others.

Differences in the types of bariatric surgery procedures are associated with differences in post-surgical weight loss. For example, AGB is primarily a restrictive procedure and is associated with the least amount of long-term weight loss among the three most popular types of bariatric surgery (i.e. SG, AGB, and RNY^20^). These post-surgical differences may also impact the efficacy of weight loss medications. Four studies have reported less weight loss for patients who have undergone SG compared to AGB^13^ and RNY^7–9^^,^^13^ from pharmaceuticals. Two of these four studies were post-hoc analysis of an original study^9^ and suggest age does not modify this relationship by observing the same pattern in younger (21–30 years)^8^ and older (≥60 years)^7^ adults. Conversely, two other studies observed similar weight loss post-initiation of pharmaceuticals for individuals who have had RYN^11^^,^^13^, and ABG^13^ or BPD^11^. Studies tended to examine a variety of medications, with no specific mention of which post-surgical patients are prescribed what medications. Thus, it could be the medication differences contributing to the findings. Pharmacokinetic studies would be beneficial to determine if physiological changes that occur with surgery impact the absorption and by extension the efficacy of weight management medications for different types of bariatric surgeries. In addition, more studies with adequate sample sizes should be conducted to explore whether there are differences in weight loss based on type of bariatric surgery when patients take the same weight management pharmaceutical.

The exclusion of post-bariatric surgery patients in phase trials has prevented the systematic testing of weight loss medication in this population. This exclusion may in part be due to concerns that changes in physiology post-surgery may make these patients susceptible to worse or different side effects from these medications^4^. Seven studies reported side effects for six medications: topiramate^4^^,^^18^, liraglutide 1.8 mg^12^^,^^15^ and 3.0 mg^14^, orlistat^17^, fenfluramine^11^, and phentermine. In general, the studies reported similar side effects in post-bariatric surgery patients as what is observed in individuals who have not had bariatric surgery. For example, GI side effects were prevalent in the studies which examined the use of liraglutide 1.8 mg^12^^,^^15^ and 3.0 mg^14^, and orlistat^17^, which is the same as non-surgical populations^21–23^. There was one unique side effect reported for post-bariatric surgery patients; memory loss with the use of phentermine^11^ which has not been reported in non-post bariatric surgical patients^24^. With the exception of phentermine, the similarities to non-surgical populations and lack severe side effects suggest that post-bariatric surgery patients may not be at an increased risk for side effects when taking weight loss medications. There are still some limitations, specifically the studies have been small in size, short in duration, and there was a novel side effect reported. Thus, large scale post marketing surveillance studies that examine different subpopulations would still be beneficial to determine if there are diverging side effects.

There are several strengths and limitations that warrant mentioning. The primary strength of this study is being the first to systematically review the literature on the use of pharmaceuticals for weight management in post-bariatric surgery patients. In addition, PRISMA, an established guideline, was used to guide the reporting of our results. Two reviewers conducted the initial search of the literature, and reviewed articles for inclusion to limit the potential impact of personal biases on article selection. Minimal restrictions were placed on retrieval criteria (e.g. no date range, no restriction on type of article, etc.), which was purposeful to increase the number of articles retrieved given the dearth of research in this area. While there was considerable heterogeneity preventing a quantitative synthesis of the findings, this also supports the notion that diverse post-bariatric surgery patients can benefit from pharmaceutical intervention. Further, despite the lack of randomized contrail trials, eleven articles received a good or high quality score using a validated bias assessment tool^5^^,^^6^.

There were limited studies comparing different types of medications, effectiveness for different types of bariatric surgery, or comparison to control groups (i.e. non-surgical, or placebo group) making it difficult to draw conclusions regarding these factors. In addition, all the articles examined reported reductions in weight regardless of the medication or bariatric surgery type. Thus, due to the absence of the studies that showed medications to be ineffective for weight loss, we cannot assess publication bias.

In summary, results of this systematic literature review suggest that patients who are prescribed pharmaceuticals for insufficient weight loss or excessive weight regain post-bariatric surgery can lose a significant amount of weight. In addition, this appears to be the case regardless of type of the pharmacotherapy. Most types of bariatric surgery examined reported similar amounts of weight loss, but there were some studies suggesting lower weight loss for patients who have had SG. Nonetheless, the side effects of the medications do not appear to differ substantially in post-bariatric surgery patients from what is typically observed in patients who have not had bariatric surgery. Taken together, this suggests that weight management medications may be an effective tool to support post-bariatric surgery patients in weight management. However, due to a lack of control, or placebo group, more rigorous research is required to determine the causal relationship between weight loss medications and changes in weight in post-bariatric surgery patients.

## Supplementary Material

Supplemental data for this article is available online at https://doi.org/10.1080/21556660.2019.1678478.

## Transparency

### Declaration of financial/other relationships

SW is the Medical Director at Wharton Medical Clinic (WMC) and an Internal Medicine specialist at Toronto East General Hospital and Hamilton Health Sciences Center. Previously, SW received payment from Novo Nordisk, Eli Lilly, Janssen, and Bausch Health for educational lectures to colleagues, as well as funding from MITACs and CIHR. EK is the current research coordinator at the WMC and works with Novo Nordisk on research projects. RAGC was employed as the Research Coordinator at the WMC, is currently working on manuscripts with Novo Nordisk, and is employed as a research consultant at WMC for works including this article. MM and AK report no conflicts of interest in this work. JDA peer reviewers on this manuscript have no relevant financial or other relationships to disclose.
