# Supplementary material for: The effectiveness and safety of pharmaceuticals to manage excess weight post-bariatric surgery: a systematic literature review
Source: J Drug Assess. 2019 Oct 17;8(1):184–91. doi: 10.1080/21556660.2019.1678478 (PMC7567517; doi:10.1080/21556660.2019.1678478)
Supplement: Supplemental data for this article is available online at https://doi.org/10.1080/21556660.2019.1678478. [file IJDA_A_1678478_SM9959.docx]

**Supplementary Table 1:** Data collection period for articles that met inclusion

| Author | Data Collection Period |
| --- | --- |
| Gorgojo-Martinez (2016)[15] | September 2011-December 2014 |
| Guerdjikova (2005)[18] | November 2002-December 2003 |
| Nor Hanipah (2018)[13] | 2012-2015 |
| Jester (1996)[11] | NR |
| Pajecki (2012)[12] | NR |
| Rye (2018)[14] | NR |
| Schwartz (2016)[16] | June 2010-April 2014 |
| Srivastava (2018)[10] | 2015-NR |
| Stanford (2017)[9] | 2000-2014 |
| Stanford (2018)[7] | 2000-2014 |
| Toth (2018)[8] | 2000-2014 |
| Zilberstein (2004)[4] | September 1999-September 2003 |
| Zoss (2002)[17] | NR |

NR, Not reported
